# Searching for New Molecular Targets for Oral Squamous Cell Carcinoma with a View to Clinical Implementation of Precision Medicine

**DOI:** 10.3390/jpm12030413

**Published:** 2022-03-07

**Authors:** Tomonori Sasahira, Miyako Kurihara-Shimomura, Yudai Shimojjukoku, Kaori Shima, Tadaaki Kirita

**Affiliations:** 1 Department of Molecular Oral Pathology and Oncology, Field of Oncology, Graduate School of Medical and Dental Sciences, Kagoshima University, Kagoshima 890-8544, Japan; yu-1059@d1.dent.kagoshima-u.ac.jp (Y.S.); kshima@dent.kagoshima-u.ac.jp (K.S.); 2 Department of Oral and Maxillofacial Surgery, Nara Medical University, Kashihara 634-8521, Japan; miyako@naramed-u.ac.jp (M.K.-S.); tkirita@naramed-u.ac.jp (T.K.)

**Keywords:** oral squamous cell carcinoma, precision medicine, biomarker

## Abstract

Head and neck cancer, including oral squamous cell carcinoma (OSCC), is the eighth most common malignancy globally and is characterized by local invasiveness and high nodal metastatic potential. The OSCC incidence is also increasing, and the number of deaths is also rising steadily in Japan. The development of molecular markers to eradicate OSCC is an urgent issue for humankind. The increase in OSCC despite the declining smoking rate may be due to several viral infections through various sexual activities and the involvement of previously unfocused carcinogens, and genetic alterations in individual patients are considered to be more complicated. Given this situation, it is difficult to combat OSCC with conventional radiotherapy and chemotherapy using cell-killing anticancer drugs alone, and the development of precision medicine, which aims to provide tailor-made medicine based on the genetic background of each patient, is gaining attention. In this review article, the current status of the comprehensive search for driver genes and biomarkers in OSCC will be briefly described, and some of the candidates for novel markers of OSCC that were found will be outlined.

## 1. Introduction

Globally, oral squamous cell carcinoma (OSCC), including lip cancer, accounts for 377,713 new diagnoses and 177,757 deaths in 2020, approximately 2% of all malignancies and 1.8% of cancer deaths [1]. In 2022, it is estimated that approximately 54,000 new patients will be diagnosed with OSCC in the United States, and 11,230 individuals will die of this disease [2]. OSCC is usually detected at an advanced stage, reflecting that it is an aggressive tumor with high local invasiveness and metastatic potential. For these reasons, the overall 5-year survival rate is <50% and has hardly improved over the past decades, despite significant advances in molecular biology and cancer treatment [3]. Recently, the number of secondary cancers seen in long-term survivors of hematopoietic stem cell transplantation has been increasing and becoming a problem, and oral cancer is the most frequent among them [4]. Additionally, OSCC often causes postoperative difficulty in chewing and swallowing, dysarthria, and loss of facial aesthetics, leading to a significant decrease in the patient’s quality of life (QOL). Therefore, the early detection and diagnosis of cancer is an urgent issue to improve the postoperative QOL of patients by suppressing OSCC and maintaining several oral functions and aesthetics.

Recently, Hanahan and Weinberg [5] proposed the concept of “Hallmarks of Cancer” that cancer acquires the following characteristics during its development and progression: (1) maintenance of growth signals, (2) evasion of growth inhibition, (3) resistance to cell death, (4) immortalization of cells, (5) invasion and metastasis, (6) angiogenesis, (7) evasion of tumor immunity, (8) abnormal energy metabolism, (9) tumor-promoting inflammation, and (10) genomic destabilization and mutation. It is generally accepted that OSCC arises from multiple genetic alterations, including deletions, insertions, point mutations, promoter methylation, gene amplification of oncogenes, inactivate tumor suppressor genes, and so on through exposure to chronic stimuli, such as alcohol consumption, smoking, certain viral infections, inflammation, ill-fitting dentures, and prostheses, as well as sharp edges of teeth because of dental caries, and these changes are acquired concurrently or heterogeneously. Among the “Hallmarks of Cancer”, invasion and metastasis are major cancer recurrence and treatment failure factors. Cancer invasion and metastasis require a series of steps: reduction in cancer cell adhesion, acquisition of motility, progression to the stroma, vascular invasion, extravasation, and proliferation at the site of metastasis [6]. Therefore, cancer should theoretically be controllable by inhibiting any of these cascades. However, identifying therapeutic targets to inhibit the invasion and metastasis of OSCC is very difficult owing to multiple genetic alterations that occur in this continuous process and the complex relationships between the individual abnormalities. 

Today, the development of diagnostic and therapeutic agents and the elucidation of the molecular pathogenesis of cancer are being actively pursued, driven in large part by remarkable advances in molecular biology and genomics. The main molecular target drugs available for oral cancer in Japan are cetuximab, a mouse monoclonal antibody targeting epidermal growth factor receptor (EGFR), and nivolumab, an immune checkpoint inhibitor that blocks the programmed death-1 (PD-1) and PD-ligand 1 (PD-L1) pathways [7]. Additionally, pembrolizumab is used to treat unresectable/metastatic OSCC with microsatellite instability-high (MSI-H) [8]. However, due to the rarity of MSI-H in oral cancer [9], the indication for pembrolizumab is very limited. Recently, entrectinib, which targets the neurotrophic receptor tyrosine kinase (*NTRK*) fusion gene, has been used across organs [10], but compared to other cancers, there are very few molecular targeted drugs available for OSCC. Thus, even with the progress made in molecular biology and genomic medicine, it is still difficult to say that there are useful diagnostic and therapeutic markers for OSCC, and identifying such markers is an urgent issue. In the future, the development of safe, effective, and highly useful drugs and diagnostics without adverse events, the establishment of biomarkers and overcoming drug resistance in oral cancer are urgently needed.

## 2. Current Status of NGS Analysis in OSCC

The development of next-generation sequencing (NGS) has enabled high-throughput analyses such as whole-genome mutation analysis, multiple target gene profiling, and comparison of tumor and normal samples. Compared with conventional polymerase chain reaction (PCR)-based genetic analysis and microarrays, NGS can provide much more detailed information in a shorter period. Of the “personalized medicine”, meaning medical treatment that selects the best treatment based on an individual’s genetic mutation and genome information, “precision medicine” is particularly concerned with finding abnormalities in biomarkers and therapeutic drugs in the vast amount of data obtained by NGS [11]. From June 2019, cancer genome profiling (CGP) test using the NGS-based FoundationOne^®^ companion diagnostic (F1CDx; Foundation Medicine, Inc, Cambridge, MA, USA) tests and OncoGuide™ NCC Oncopanel System (Sysmex Corp, Kobe, Japan) will be covered by insurance in Japan for patients with solid tumors who have not received or completed standard treatment [12]. The CGP test is used to determine the optimal molecular targeted drugs that match the genetic mutations and genomic information of each patient, and its widespread use is expected to accelerate the development of precision medicine in the future. However, most of the targeted drugs that match the genetic abnormality are administered through participation in clinical trials or off-label use, and only a few percent to a dozen percent of patients who undergo CGP testing receive treatment based on the detected genetic mutation [13,14]. Although there are still many problems to be solved at this point, it is undeniable that this is a revolutionary test for finding new treatment methods, and it is expected to bring great benefits to cancer patients who have run out of options.

**Figure 1:**
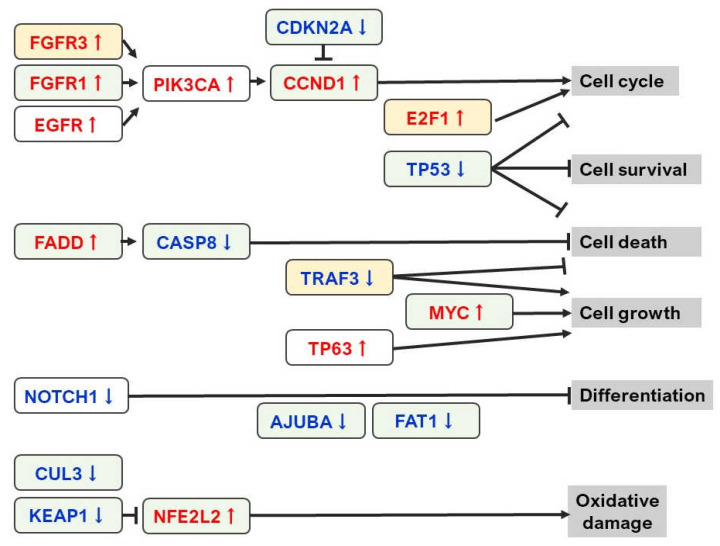
Shema of signaling pathways highly prevalent in head and neck cancer. The green and yellow columns represent HPV-negative and HPV-positive cancer predominance, respectively. Figure in [15] was modified.

Recently, a report on large-scale analysis of genetic abnormalities in head and neck SCC (HNSCC), including OSCC, using NGS was published [15]. The outline of the genetic abnormality is summarized in Figure 1. According to the report, cyclin-dependent kinase inhibitor 2A (*CDKN2A*), cyclin D1 (*CCND1*), *EGFR*, fibroblast growth factor receptor 1 (*FGFR1*), Fas-associated via death domain (*FADD*), FAT atypical cadherin 1 (*FAT1*), tumor protein 53 (*TP53*) caspase-8 (*CASP8*), Notch receptor 1 (*NOTCH1*), ajuba LIM protein (*AJUBA*), and MYC proto-oncogene (*MYC*) genotypes are the most important in human papilloma virus (HPV)-negative HNSCC. Among them, *CASP* 8 mutation was observed in 92% of OSCC, and cases with HRas Proto-Oncogene, GTPase (*HRAS*) and phosphatidylinositol-4,5-bisphosphate 3-bisphosphate catalytic subunit alpha (*PIK3CA*) activation, *NOTCH1* inactivation, and wild-type *TP53* have particularly had a favorable prognosis. Regarding the association with smoking, which is a major cause of oral carcinogenesis, *TP53* mutation, *CDKN2A* loss of function, amplification of 3q and 11q, and alterations of oxidative stress-related genes Kelch-like ECH-associated protein 1 (*KEAP1*), nuclear factor, erythroid 2 like 2 (*NFE2L2*), and cullin 3 (*CUL3*) has been clarified. This report also suggests that a single driver mutation does not cause HPV-positive group, although they tend to show TNF receptor-associated factor 3 (*TRAF3*) inactivation and amplification of *FGFR3* and *E2F1*, and fewer mutations in *TP53*, *CDKN2A,* and nuclear receptor binding SET domain protein 1 (*NSD1*).

As shown in Table 1, HPV-positive oropharyngeal carcinoma (including OSCC) has different biological characteristics from the usual OSCC [16]. Although radiation therapy with cetuximab was expected to be effective in HPV-related oropharyngeal cancer [17], recent reports have revealed that the efficacy of this combination therapy is poor, and the combination of cisplatin and radiation therapy is the current standard treatment [18,19].

At present, attempts to classify cancers into molecular subtypes based on gene expression profiles are being made, and HNSCC has been classified into basal (BA), atypical, mesenchymal, and classical molecular subtypes based on differences in the status of the *KEAP1* and *NFE2L2* oxidative stress pathway, expression of the lineage markers SRY-box transcription factor 2 (*SOX2*) and TP63, and activation of the oncogenes *PIK3CA* and *EGFR* (Table 2) [20]. Keck et al. [21] have also reported that HNSCC is classified into five subtypes: BA, CL-HPV, CL-non-HPV, inflamed/mesenchymal subtypes (IMS)-HPV, and IMS-non-HPV. According to their study, the IMS subtype is involved in tumor immunity, and the two HPV subtypes are characterized by low expression of EGFR/HER ligands and no copy number events. The BA subtype, on the other hand, is characterized by a prominent EGFR/HER signaling phenotype, HPV negativity, and strong hypoxic differentiation. In addition, transcriptome profiling of approximately 6000 single cells from 18 HNSCC patients demonstrated that the expression of signatures related to cell cycle, stress, hypoxia, epithelial differentiation, and partial epithelial-mesenchymal transition (p-EMT) are important in malignant cells, and p-EMT, in particular, is a predictor of lymph node metastasis and malignancy in cancer [22]. Huang et al. [23] also classified HNSCC with lymph node metastasis into molecular subtypes of Group 1 (immune subtype with enriched pathways, including allograft rejection, T-cell receptor signaling pathway, and chemokine receptor signaling), Group 2 (invasive subtype with enriched pathways, including epithelial–mesenchymal transition, apical junction, angiogenesis, hypoxia, extracellular matrix receptor interactions, regulation of the actin cytoskeleton, and focal adhesion), and Group 3 (metabolic/proliferative subtype with enriched pathways, including MYC targets, basal transcription factors, and mismatch repair) based on comprehensive mRNA expression profiles. Among these subtypes, Group 2 has the worst overall survival rate and disease-free survival rate, as well as the lowest local control rate of cancer. Identifying molecular subtypes of OSCC based on such comprehensive analysis will greatly contribute to the selection and development of optimal cancer therapeutics agents for individuals.

## 3. Tumor Biomarker and Liquid Biopsy

Although there have been many studies on tumor biomarkers, practical and useful ones for OSCC have not yet been established. Despite the importance of postoperative adjuvant therapy along with surgery to treat advanced OSCC, conventional methods, such as chemotherapy using cell-killing anticancer drugs, radiation therapy, and combination therapy of both still remain the mainstream of adjuvant therapy for OSCC. These conventional methods also inevitably cause significant harm to normal cells. The current situation is that there are almost no definitive treatment options. In the coming era, it is expected that the development of clinical tests (diagnostic markers) based on gene and protein expression and mutation profiles, prediction of metastatic potential and prognosis will be actively promoted. A comprehensive analysis of genomic data is also useful for molecular biological cancer diagnosis and subclassification. Furthermore, the accumulation of genetic information related to carcinogenesis and cancer progression will directly lead to the development of effective new drugs by clarifying mutated genes and the signaling pathways altered by them.

Tumor biomarkers are divided into several types: screening markers, early diagnostic markers, staging markers, prognostic markers, and predictive and monitoring markers [24]. It has been reported that tumor biomarkers must meet the following conditions. (1) the change must be objectively quantifiable, (2) it must be possible to measure with a small amount of sample, (3) the change must occur in cancerous tissue, not in normal tissue, and (4) it must be detected in the early stages of cancer development [25]. Presently, attempts at diagnosis by liquid biopsy using blood, urine, saliva, pleural fluid, ascites, etc. have been gaining attention. The information obtained by liquid biopsy reflects changes throughout the tumor and has the advantage of being less susceptible to tumor heterogeneity compared to tissue biopsy. It is also less invasive, results can be obtained quickly, and biomarker monitoring is possible [26]. The main targets for biomarker discovery by liquid biopsy using blood are cell-free DNA (cfDNA), circulating tumor cells (CTC), and microRNA (miR, miRNA), which are shown below.

### 3.1. cfDNA

Circulating cfDNA is released into the blood due to cell apoptosis or necrosis, and those derived from tumor cells are especially called circulating tumor DNA (ctDNA) [27]. Since cfDNA is fragmented by endonuclease, the average cfDNA concentration in the blood of healthy people is about 10–15 ng/mL, but the blood ctDNA of patients with cancer is often highly concentrated [27,28]. The major clinical application of ctDNA analysis is the identification of new mutations by gene profiling and selecting effective molecular targeted drugs [29]. Previous reports have shown that *TP53*, *PIK3CA*, *CDKN2A*, F-box, and WD repeat domain containing 7 (*FBXW7*), *HRAS*, *NOTCH1*, *CASP8*, and phosphatase and tensin homolog (*PTEN*) mutations can be detected in plasma ctDNA [15,30]. It was also reported that hypermethylation of short stature homeobox protein 2 (*SHOX2*) and septin 9 (*SEPT9*) in ctDNA is observed in 59% of patients with HNSCC with 96% specificity [31]. Therefore, ctDNA in blood can be a useful OSCC biomarker.

### 3.2. CTC

CTCs are circulating cells released from tumor tissue into the bloodstream and play a critical role in the formation of metastases [27,32]. Many of the CTCs have the ability to leave the primary tumor lesion by acquiring epithelial–mesenchymal transition (EMT) phenotypes and eventually form new metastases [32]. Previous reports have shown that HNSCC patients with lymph node metastasis have a significantly higher detection rate of CTCs in their blood than those without [33]. Global gene expression analysis by microarray using blood CTC from 36 patients with OSCC has shown that upregulation of *CD274* (commonly known as PD-L1), homeobox B9 (*HOXB9)*, and zinc finger protein 813 (*ZNF813*) and decreased expression of B cell linker (*BLNK*) are frequently observed [34]. In relation to treatment, the number of CTCs in the blood has been found to be significantly reduced after chemoradiation therapy in OSCC [35]. It was also shown that HNSCC patients who responded to nivolumab treatment had higher levels of *CCND1* expression in their CTCs [36]. Additionally, phosphorylation levels of EGFR are dramatically reduced in the blood CTCs of patients with OSCC who responded to cetuximab [37]. In addition, there are reports that the presence of CTCs in HNSCC contributes to prognosis in HNSCC patients [36,38,39], and in particular, patients with CTCs expressing *MET* have a significantly poorer prognosis [36]. Thereby, clarifying the molecular characterization of CTCs may be useful for predicting the potential for metastasis, successful treatment, and clinical outcome in OSCC. Since recent studies have revealed that CTCs have different characteristics from both primary tumors and metastatic tumors [38]. CTCs are expected to be applied to many fields, including understanding metastatic mechanisms, searching for new therapeutic targets, and developing personalized medicine. However, reliable CTC capture and improved single-cell sequencing are essential for clinical implementation, and further system development is expected [32].

### 3.3. miRNA

Exosomes are vesicles secreted by cells and contain mRNA, genomic DNA, cDNA, miRNA, etc. It has long been believed that gene functions are brought about by the proteins they code, and non-coding RNA (ncRNA), which do not code for specific proteins, were not considered important as “junk in the genome”. However, it has become clear that ncRNA also plays an important role, especially miRNA, a small single-stranded RNA of approximately 18–25 nucleotides that negatively regulate gene expression by binding to the 3’ untranslated region (UTR) of target genes with complementary sequences [39]. The biosynthetic process of the mature miRNA can be explained as follows: Primary miRNA (pri-miRNA) is converted to precursor miRNA (pre-miRNA) by RNase Drosha and DiGeorge syndrome critical region gene 8 (*DCRG8*) in the nucleus. The pre-miRNA is transported into the cytoplasm by exportin 5 and processed into mature microRNA by RNase Dicer. Mature miRNA regulates mRNA expression of target genes after being incorporated into the RNA-inducible silencing complex (RISC) [6]. In recent studies using miRNA arrays, upregulation of *miR-19a*, *miR-512-3p*, *miR-27b*, *miR-20a*, *miR-28-3p*, *miR-200c*, *miR-151-3p*, *miR-223*, and *miR-20b* and decreased expression of *miR-22*, *miR-370*, *miR-139-5p*, *let-7e*, *miR-145-3p*, and *miR-30c* have been confirmed in exosomes of patients with OSCC [40]. Additionally, plasma levels of *miR-31* and *miR-184* have decreased after tumor resection and are expected to be useful markers of disease progression [28,41,42]. MiRNA molecules in exosomes can be a minimally invasive test marker, and it is necessary to further clarify their functional significance in the future.

## 4. Candidate for Novel Secretory Molecular Markers in OSCC

Despite NGS development and the search for OSCC molecular markers, as aforementioned, there are still numerous issues that need to be addressed. The molecular classification of OSCC and identification of driver genes are extremely difficult. Additionally, there are still no biomarkers or molecularly targeted drug candidates to be used as a decisive factor in OSCC. Several candidate molecules involved in the characterization of OSCC were revealed. Section 3 and Section 4 outline the recently identified new markers separately for secretory and non-secretory components, respectively.

### 4.1. TANGO and Related Molecules (MUC20 and SRPX2)

#### 4.1.1. TANGO

Members of the melanoma inhibitory activity (*MIA*) gene family are secreted proteins that comprise *MIA*, *MIA2*, transport and Golgi organization protein 1 (*TANGO*), and otoraplin (*OTOR*) and share 34–45% amino acid homology and 47–59% cDNA sequence homology and possess a highly conserved Src homology 3 (SH3)-like domain and hydrophobic N-terminal secretory signal sequence [43]. Among them, *TANGO* interacts with integrin CD11c/CD18 and influences premonocytic cells by reducing attachment and promoting migration [44]. *TANGO* is also a tumor suppressor in malignant melanoma [43] and colorectal and hepatocellular carcinoma [42]. Conversely, it was found that *TANGO* promoted cell proliferation and invasion and inhibited apoptosis in OSCC cells [45]. Gene expression levels of *TANGO* were higher in almost all OSCC cases and cells than in the normal oral mucosa and other cancer cells and specimens. Additionally, *TANGO* regulated the adhesion of OSCC cells to primary human umbilical vein endothelial cells (HUVEC) and primary human dermal lymphatic microvascular endothelial cells (HDLMVEC) and transendothelial migration and tube formation of endothelial cells by activating platelet-derived growth factor beta polypeptide (*PDGFB*) and neuropilin 2 (*NRP2*). Upon immunostaining with OSCC material, the positive rate of *TANGO* was 35.1% (60/171), and its expression was associated with T stage, clinical stage, lymph node metastasis, microvessel density (MVD), lymphovessel density (LVD), and expression of *PDGFB* and *NRP2*. Additionally, cases expressing *TANGO* had a significantly poorer prognosis. *TANGO* may be a new biomarker of angiogenesis and lymphangiogenesis in OSCC.

*TANGO* and other members of the *MIA* gene family are secreted proteins that are detected at high levels in the serum and saliva of oral cancer patients, and MUC20 is probably secreted and detected in the same way. In actual clinical practice, measuring the secretion of these factors in saliva will have great potential for application as a minimally invasive tumor marker. For more information on the *MIA* gene family in OSCC, please refer to my previous review [46].

#### 4.1.2. MUC20

Since differences in downstream signaling were thought to be the reason why *TANGO*, a tumor suppressor in other cancers, functions in a pro-tumor manner in OSCC, microarray analysis was performed using multiple OSCC cells, and mucin 20 (MUC20) was identified as a new molecule located downstream of *TANGO* [47]. MUC20 is a molecule involved in mucin production and has been reported to play a tumor-promoting role in gastrointestinal cancer [48,49] and gynecological cancer [50]. Expression levels of MUC20 were increased or decreased in OSCC cells treated with *TANGO* gene transfer or knockdown, respectively. Furthermore, expression levels of MUC20 were decreased or restored by *TANGO* antibody treatment or recombinant protein addition to OSCC cells, respectively. These results indicate that *TANGO* activates MUC20*TANGO*. In MUC20-transfected cell lines, interaction with *TANGO* enhances proliferative potential through increased c-met phosphorylation and contributes to invasive potential by decreasing the secretion of E-cadherin, which is involved in cell adhesion, and increasing the secretion of matrix metalloproteinase-2 (MMP-2), which promotes stromal destruction. MUC20 also enhanced the migration, proliferation, and luminal formation of HUVEC and HDLMVEC via VEGF-A and VEGF-C, as well as the adhesion between OSCC and endothelial cells. Real-time reverse transcription-polymerase chain reaction (RT-PCR) analysis using 30 frozen specimens of OSCC showed higher gene expression levels of MUC20 than the normal oral mucosa. In immunohistochemistry of formalin-fixed paraffin-embedded (FFPE) specimens of OSCC cases, MUC20 expression (64/213; 30.1%) was significantly correlated with clinical stage, lymph node metastasis, MVD, LVD, and poor prognosis. These results suggest that MUC20, whose expression is regulated by *TANGO*, is involved in OSCC progression.

In esophageal SCC, MUC20 has been reported to be involved in the acquisition of paclitaxel resistance [51]. Paclitaxel is an anticancer drug commonly used in OSCC, and MUC may be useful as a biomarker to predict anticancer drug resistance in OSCC. Additionally, since MUC20 is a secreted protein, it is expected to be clinically applicable as a minimally invasive and monitorable biomarker.

#### 4.1.3. SPRR1B

**Figure 2:**
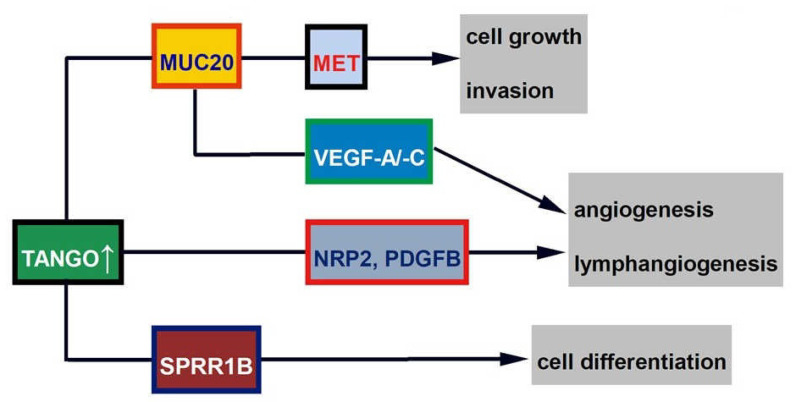
Shema of *TANGO*-related signal.

Small proline-rich protein 1B (*SPRR1B*) is another novel molecule downstream of *TANGO* in OSCC [47]. *SPRR1B* is a marker for squamous cell differentiation, and its expression is high in the tongue, skin, and well-differentiated cutaneous and esophageal SCC [52]. *SPRR1B* is also associated with squamous metaplasia of the airway epithelium [53], and its expression is lower in gastric cancer [54] and lung adenocarcinoma [55]. In clinical samples, expression levels of *SPRR1B* were significantly higher in adjacent normal mucosa than in OSCC tissues. Moreover, *SPRR1B* immunopositivity was 71.6% (88/123) in well-differentiated OSCC but 53.3% (48/90) in those with moderate to poorly differentiated OSCC. In an in vitro study using OSCC cells, well-differentiated HSC4 cells expressed higher levels of *SPRR1B* than poorly differentiated HSC3 cells. It was also found that the interaction of *TANGO* with *SPRR1B* increased the expression of involucrin and keratin 1, which are differentiation markers of keratinocytes. It has been reported that MAPK p38 plays an important role in keratinocyte proliferation [56], and it was also found that *SPRR1B* induces MAPK p38 proliferation and promotes proliferation of well-differentiated OSCC cells. These results suggest that *SPRR1B* is involved in OSCC cell differentiation. The schema of *TANGO*-related signals is shown in Figure 2.

Since involucrin and keratin 1 are thought to be the molecules involved in the final differentiation of keratinocytes [57], *SPRR1B* may contribute to the final determination of OSCC differentiation in the presence of *TANGO*. In poorly differentiated OSCC, *SPRR1B* transfection may reduce malignancy by stimulating the proliferation of mature keratinocytes through MAPK p38 phosphorylation and increasing the degree of differentiation. The usefulness of *SPRR1B* as a therapeutic target in OSCC needs to be further investigated.

### 4.2. LEMD1 and Ralated Molecules (SRPX2 and SERPINE2)

#### 4.2.1. LEMD1

LEM domain containing 1 (LEMD1) is a type of cancer-testis antigen (CTA) whose expression is highly restricted to testicular germ cells and has been reported to be highly expressed in colorectal cancer [58]. In immunohistochemistry using 289 OSCC specimens, the LEMD1 positivity rate was 35% (101/289), and its expression was significantly correlated with tumor progression (T stage and clinical stage) and nodal metastasis [24]. Multivariate analysis using the COX proportional hazards model revealed that LEMD1 expression was an independent poor prognostic factor for OSCC. In vitro studies showed that LEMD1 promoted invasion of OSCC cells, adhesion of OSCC cells to HUVEC and HDLMVEC, and migration and tube formation of endothelial cells. Although further studies are needed, these results indicate that LEMD1 is a novel biomarker for OSCC.

LEMD1 is a CTA. Recently, oncogenic CTA in malignant tumors has been suggested to be useful targets for immunotherapy by activating cytotoxic T lymphocytes [59]. The normalization of LEMD1 may be useful for activating the host immune function, but further in vivo and in vitro studies are required to clarify the possibility of LEMD1-targeted immunotherapy.

#### 4.2.2. SRPX2

Sushi repeat-containing protein X-linked 2 (*SRPX2*) is a gene involved in the development of the language centers of the brain (Broca’s area and Wernicke’s area) [60] and has tumor-promoting functions in gastrointestinal cancer [61,62,63]. *SRPX2* was found to be a novel gene related to *LEMD1* by a comprehensive gene expression analysis using microarray [64]. As receptors for *SRPX2*, urokinase plasminogen activator receptor (uPAR) [65] and hepatocyte growth factor (HGF) [61] are known, and it was found that *SRPX2* was secreted into the culture supernatant by treating oral cancer cell lines with recombinant proteins of uPAR and HGF. Additionally, cell culture of vascular and lymphatic endothelial cells in supernatants of OSCC cells that highly express *SRPX2* increases migration and proliferation, but these effects are attenuated by combined treatment with anti-uPAR and anti-HGF antibodies. Therefore, *SRPX2* may be a ligand for uPAR and HGF in OSCC. Notably, *SRPX2* is associated with anticancer drug resistance and was closely related to the development of resistance to platinum drugs such as cisplatin and nedaplatin in OSCC cells. The positive rate of *SRPX2* in immunostaining of 161 FFPE specimens was 28% (45/161), and *SRPX2* was more highly expressed in patients with lymph node metastasis or high MVD or LVD, and those with high *SRPX2* expression had a significantly poorer prognosis than those without. Additionally, the expression levels of *LEMD1* and *SERPINE2* correlated well with each other in real-time RT-PCR using frozen OSCC specimens.

To improve OSCC prognosis, early detection and effective adjuvant therapy after surgery are extremely important. If effective adjuvant therapy can be selected, it will be possible to preserve oral functions (speech, pronunciation, mastication, swallowing, etc.) and maintain facial aesthetics after surgery. However, there are currently no definitive chemotherapy, radiotherapy, or chemoradiotherapy resumes in patients with OSCC. Since *SRPX2* is a secreted protein and is resistant to platinum drugs, measuring its secretion levels in saliva may be applied to predict the effect of adjuvant therapy and select appropriate anticancer drugs.

#### 4.2.3. SERPINE2

**Figure 3:**
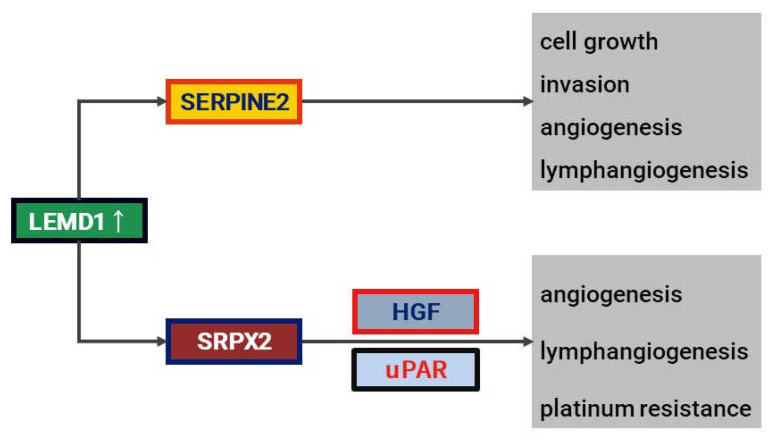
Shema of LEMD1-associated signal.

Serpin peptidase inhibitor, clade E, member 2 (*SERPINE2*), has been reported to be a risk gene involved in OSCC development in genome-wide haplotype analysis [66], but it acts as a tumor suppressor in other cancer types [67,68,69]. Therefore, its function is not well understood. *SERPINE2* is another molecule that the authors found as a LEMD1-related signal using microarray analysis [70]. It was confirmed that knockdown of the SERPINE2 gene in OSCC cells suppressed the expression of SERPINE2 at the protein level and the secretion into the culture supernatant. In 42 OSCC samples, higher levels of *SERPINE2* secretion than in normal mucosa were confirmed by ELISA, indicating that *SERPINE2* acts as a secreted protein in OSCC. Additionally, the effect of *SERPINE2* on angiogenesis was investigated in vitro, and it was found that *SERPINE2* induced proliferation, migration, and tube formation of HUVEC and HDLMVEC and promoted the interaction between OSCC and endothelial cells. Furthermore, *SERPINE2* enhanced proliferation and invasion ability in OSCC cells. In immunohistochemistry of 167 FFPE specimens, the *SERPINE2* expression rate was 38.9% (65/167), and its expression was correlated with the depth of invasion, MVD, and LVD. Additionally, it was shown to be an independent poor prognostic factor in multivariate analysis. These results suggest that *SERPINE2* acts as a vascular/lymphangiogenic factor in oral cancer by exerting its function as a secreted protein. The downstream signal of LEMD1 is shown in Figure 3.

Tumor blood vessels/lymphatic vessels supply nutrients to cancer, but compared to normal blood and lymphatic vessels, they have an irregular morphology with no pericyte lining and insufficient lumen formation, making it difficult for anticancer drugs to reach the cancer site [71]. In addition, tumor blood vessels continue to produce VEGF family members due to continued hypoxia because of their irregular structure and very poor blood flow. Furthermore, the increased vascular permeability increases the pressure in the tumor stroma, suggesting a vicious cycle that reduces the efficiency of anticancer drug transfer from blood vessels to the tumor [72]. The inhibition of tumor angiogenesis and/or lymphangiogenesis may dramatically reduce cancer progression. Anti-angiogenic drugs are frequently used in cancer types other than OSCC, but unfortunately, there are currently no anti-angiogenic molecular target drugs approved by insurance for OSCC in Japan. Recently, a new concept of anti-angiogenic and/or lymphangiogenic therapy has been proposed to normalize abnormal blood and lymphatic vessels to improve local delivery and retention of anticancer drugs [73,74]. The possibility of anti-angiogenic/lymphangiogenic therapy targeting *SERPINE2* is also expected, and further investigation is needed in the future.

## 5. Candidate for New Non-Secretory Markers in OSCC

### 5.1. PXDN

Cancer cells suppress mitochondrial activity, shift glucose metabolism primarily to glycolysis, and produce lactate and adenosine triphosphate (ATP) even in the presence of oxygen (Warburg effect). These metabolic changes are thought to induce the generation of reactive oxygen species (ROS) and promote cancer progression [75]. Recent reports suggest that peroxidasin (*PXDN*) promotes tumorigenesis and the survival of prostate cancer cells by inhibiting oxidative stress through the removal of ROS and inducing apoptosis through the Warburg effect [76]. *PXDN* overexpression was observed in various malignancies [77,78]. In OSCC, *PXDN* mRNA upregulation was confirmed in 42 of 111 cases (37.8%) and was also expressed at higher levels in cases with lymph node metastasis and diffuse invasion patterns. Patients with high *PXDN* expression had a significantly poorer prognosis, and a high *PXDN* expression was an independent poor prognostic factor in multivariate analysis. Furthermore, expression levels of *PXDN* in OSCC were proportional to lactate and ATP production and inversely correlated with ROS production but not with the activation state of mitochondria. These results suggest that *PXDN* might promote OSCC by affecting cancer cell metabolism [79].

Generally, the Warburg effect on cancer cells is believed to promote cancer invasion and metastasis [75]. Conversely, the Warburg effect acts in a tumor-suppressive manner in the early stages of carcinogenesis [80]. Although there are many unanswered questions about the Warburg effect in cancer, these results suggest that the Warburg effect induced by *PXDN* is cancer-promoting. Since ROS produced by defective mitochondria promotes cancer development and progression, reducing oxidative stress was considered a potential means of cancer prevention and treatment [81]. However, there are reports that antioxidants promote the development and metastasis of cancer [82,83]. Furthermore, it has been reported that the accumulation of excess ROS in tumor cells inhibits cancer progression by arresting cancer growth and inducing cell death [84], while CD44-mediated suppression of intracellular ROS has been shown to result in resistance to cancer therapy [85]. Thus, ROS has a double-edged function in cancer. *PXDN* may contribute to cancer progression by regulating ROS production to low levels.

### 5.2. miR-29b-1-5p

Among miRNA, *miR-29b-1-5p* is attenuated in the expression in triple-negative [86] and basal-like breast cancer [87], whereas it has been reported that overexpression is involved in the acquisition of proliferative ability in bladder cancer [88], and no consensus has been obtained. Laser capture microdissection (LCM) was used to isolate only cancer cells from FFPE specimens of 49 OSCC cases, and then, real-time RT-PCR was performed. The results showed that *miR-29b-1-5p* was highly expressed in cases showing EMT. Furthermore, miR-29b-1-5p overexpressing cases had a significantly poorer prognosis than those who did not. EMT is a phenomenon in which the infiltration capacity is strengthened by showing a decrease in the E-cadherin (CDH1) expression involved in cell adhesion and an increase in vimentin (VIM) expression, which is a mesenchymal marker, in cancer [89]. Among OSCC cells, HSC4 cells with EMT-negative phenotype show low expression of *miR-29b-1-5p*, whereas KON cells with EMT-positive phenotype show high expression of *miR-29b-1-5p*. *MiR-29b1-5p* has a complementary nucleotide sequence to the 3′-UTR of *CDH1*, and modulation of *miR-29b-1-5p* expression altered luciferase activity, whereas mutation of the 3′-UTR of *CDH1* abolished its function. Additionally, transfection with *miR-29b-1-5p* resulted in the acquisition of EMT traits such as decreased CDH1 expression and increased VIM expression in HSC4 cells, whereas the repression of *miR-29b-1-5p* reduced the EMT characteristics in KON cells. These results indicate that *miR-29b-1-5p* is involved in EMT by targeting *CDH1* in OSCC [90].

LCM is a method to collect only the target cells from FFPE specimens and is ideal for studying cancer cell-specific gene expression from biological samples. Nucleic acids in FFPE specimens are highly cross-linked and often degraded, whereas miRNA is a small molecule and is likely to maintain its expression even in FFPE specimens with some long shelf life [91]. Furthermore, as mentioned above, miRNA is thought to be contained in saliva, and a minimally invasive test can be performed by measuring the expression level in saliva. It is a molecule that is greatly expected to be clinically applied to diagnosis and treatment.

## 6. Future Perspectives

Saliva can be an ideal laboratory material for OSCC because it is noninvasive, economical, easy to collect, repeatable, collectible not only by physicians and dentists but also by patients themselves and their families, and can be monitored. Since saliva is in direct contact with OSCC, it is highly anticipated to contain several biomarkers. It has been reported that hypermethylation of tissue inhibitors of metalloproteinase 3 (*TIMP3*), *CDH1*, *CDKN2A*, O-6-methylguanine-DNA methyltransferase (*MGMT*), death-associated protein kinase (*DAPK*), and Ras association domain family member 1 (*RASSF1*) could be detected in salivary DNA of 90 patients with HNSCC [92]. Furthermore, DNA methylation levels of *RASSF1*, *CDKN2A*, *TIMP3*, and mediator complex subunit 15 (*MED15*) in salivary DNA of HPV-negative HNSCC patients are higher than those of HPV-positive patients and healthy individuals [93]. Exosome analysis in saliva was reported to detect *miR-302b-3p* and *miR-517b-3p* only in extracellular vesicles of patients with OSCC, suggesting that these microRNAs may be useful as metastasis monitoring markers [94]. There are high expectations for the usefulness of saliva as a tool to search for OSCC biomarkers.

Artificial intelligence (AI) is being applied to the field of cancer diagnosis and treatment, and there is no doubt that this trend will accelerate in the future. Toward the clinical application of precision medicine, it is highly expected that new target genetic abnormalities will be discovered by analyzing the vast amount of information accumulated in the database with the maximum use of AI, leading to the development of innovative new drugs and diagnostic methods. With further progress in identifying driver genes for several cancers and developing modalities that can detect gene abnormalities with higher accuracy, it can be asserted that precision medicine will play a central role in cancer medical care in the near future.

## 7. Conclusions

Current pathological diagnosis is based on our predecessors’ vast amounts of morphological knowledge. Conversely, it is also true that morphology alone is not enough to face the complex and fragmented pathology of today, and the fields of molecular pathology and genomic pathology will become increasingly important in the future. It is easy to imagine that the morphological changes seen in cancer may largely reflect individual genetic mutations, and future pathologists will be required to have the ability to capture the signals of genetic abnormalities emitted by cancer cells under the microscope. In cancer pathology, morphology and molecules are inseparable. As mentioned above, it is not easy to narrow down the candidates for molecular targets because of the complex effects of several genetic changes during the development, progression, and metastasis of OSCC. Most of the genetic abnormalities that were identified are thought to be passenger mutations that occur incidentally to the development of OSCC, and unfortunately, we have not yet been able to find the driver genes. We have also not yet reached the point of clinical application of molecular diagnosis and treatment targeting these novel factors. However, identifying driver genes is essential for the control of OSCC, and cancer pathologists with an eye for molecules and the ability to diagnose through morphology will play an important role. An understanding of the genetic diversity among individuals and information on genetic alterations (germline and somatic) and epigenetic changes in the process of carcinogenesis and cancer progression is essential to the practice of precision medicine, which determines each patient’s optimal treatment. Precision medicine will revolutionize clinical oncology and oncological pathology. Further development of precision medicine in OSCC is expected in the future.

## Figures and Tables

**Table 1:** Biological features of head and neck cancer associated with HPV infection.

	HPV-Positive Cancer	HPV-Negative Cancer
Risk factor	Oral sex	Smoking, alcohol abuse
Age	Under 60 years	Above 60 years
Surrogate marker	p16	p53
Susceptible site	Oropharynx	Various sites
Outcome	Good	Bad

**Table 2:** Biological characteristics of head and neck cancer by molecular subtype.

	Basal	Mesenchymal	Atypical	Classical
Differentiation	Well, moderate	Moderate, poor	Moderate	Moderate, poor
Predominant site	Oral cavity	Oral cavity	Oropharynx	Larynx
Lymph node metastability	Low	Moderate	High	Low
7p gain	Yes	Yes	No	Yes
*CDKN2A* loss	Occasionally	Rarely	Rarely	Frequently
*SOX2* expression	Low	Low	High	High
*TP63* expression	High	Moderate	Moderate	Moderate

## Data Availability

Not applicable.
